# Effects of the COVID-19 pandemic on maternal, newborn, and child health service coverage in Burkina Faso

**DOI:** 10.7189/jogh.14.05037

**Published:** 2024-12-20

**Authors:** Abdoulaye Maïga, Moussa Bougma, Emily Wilson, Théodore S Kaboré, Gildas G Tou, Melinda K Munos, Almamy M Kanté, Safia S Jiwani, Kelsey Zack, Aveika Akum, Neff Walker, Robert E Black, Agbessi Amouzou

**Affiliations:** 1 Department of International Health, Johns Hopkins University Bloomberg School of Public Health, Baltimore, Maryland, USA; 2 Institut Supérieur des Sciences de la Population, Université Joseph KI-ZERBO, Ouagadougou, Burkina Faso

## Abstract

**Background:**

While countries’ coronavirus disease 2019 (COVID-19) emergency contingency and response plans aimed to prevent and control the spread of the virus, they also caused major disruptions to health services. We assessed the effects of COVID-19 on coverage and inequalities in select maternal, newborn, and child health services in Burkina Faso.

**Methods:**

We analysed data from two cross-sectional household surveys conducted in two provinces, one rural and one urban. The first survey of 3375 households was conducted immediately before the pandemic (February to March 2020) and the second survey in the same areas two years after the pandemic (May to June 2022) using a similar methodology. We compared the coverage of maternal, newborn, and child health interventions and care-seeking between the two surveys to assess the effects of the pandemic on maternal, newborn, and child health services.

**Results:**

Our findings did not show significant disruptions in coverage of antenatal service, postnatal care for mothers and babies, child routine vaccination, and care-seeking for sick children during the pandemic. However, there was a dramatic drop of the number of women (23 percentage points) accompanied by their partners for delivery as well as the number of caesarean-section deliveries in urban areas. The shortage of health staff, facility congestion, fear of getting COVID-19 after a caesarean-section admission, and prioritisation of critical health services such as emergency caesarean-section to the detriment of elective cases may explain the decline of caesarean-section rates.

**Conclusions:**

COVID-19 did not cause major reversals in the coverage of maternal, newborn, and child health services in Burkina Faso, except for caesarean sections. We also saw no substantial increases in service coverage. In the absence of a counterfactual, we could not attribute the stagnation to the pandemic. However, the very low proportion of women reporting disruption in care-seeking suggests some resilience of the health systems to mitigate the negative impacts of the pandemic.

Early warnings and estimates predicted a stronger negative impact of the coronavirus disease 2019 (COVID-19) pandemic in low- and middle-income countries (LMICs) due to weak health systems and poor social conditions [1–6]. Several forecasts predicted reduced coverage of essential health services and population mortality rates. An earlier modelling of the indirect effects of COVID-19 suggested that LMICs would experience an increase of up to 39% in maternal and 45% in child deaths per month [2]. This would represent 56 700 additional maternal deaths and 1 157 000 additional child deaths over six months across 118 countries [2]. As of 20 December 2020, 1.7 million cumulative cases and 38 000 deaths were reported in African countries, representing 2% for both global cases and deaths, while this region accounts for 17% of the world’s population [7,8]. Most countries followed the World Health Organization (WHO) recommendations by activating their epidemic emergency contingency measures and policies, preparedness, and response plans. These included containment measures to prevent and control the spread of the pandemic such as temporary lockdowns, closing of schools and churches, ban of social gatherings, enforcement of facemask wearing and other personal protective equipment and measures [9,10]. The health workforce and service provision were also reorganised to isolate and manage the COVID-19 cases.

While these measures were designed to prevent or minimise the risk of transmission of the virus, they also had potentially serious inhibiting consequences on health service utilisation for acute and chronic care, not only in terms of disruption to health and nutrition services availability but also due to fears of nosocomial infections and increased physical and financial barriers to access to services [11]. These indirect effects were expected to cause major reversals in hard-earned gains in reproductive, maternal, newborn, and child health and nutrition services coverage, as well as with mortality and malnutrition rates, in countries with fragile and vulnerable health systems [4,12,13]. Lessons from the Ebola epidemic in West Africa suggest that the indirect effects of pandemics could be devastating for reproductive, maternal, newborn, and child health and nutrition, socioeconomic status, and social cohesion [14]. For example, a scoping review on the indirect effects of the Ebola epidemic in West Africa showed up to 80% decline in maternity care, a 40% reduction in malaria admission in children under five and a reduction in child immunisation in highly-affected areas [15].

Numerous initiatives and studies tried to measure the indirect effects of the pandemic by relying on readily available data from health information systems and modelling based on assumed declines in intervention coverage [2,4,16,17]. The models predicted a decline of more than 50% of reproductive, maternal, newborn, and child health service coverage during the earlier periods of the COVID-19 lockdown that may result in a 22% increase in maternal deaths and 14% stillbirths in LMCIs [18]. Other predicted effects suggested a 14.3% increase in child wasting due to the disruption of food distribution systems and a decrease in the gross national income per capita [6]. The potential catastrophic disruption to food security systems, as well as social protection and its impact on acute and chronic malnutrition, prompted leaders of key United Nations agencies to call for the implementation of several urgent actions to protect children’s right to nutrition during the pandemic [5].

Many previous studies used routine health information systems data to assess the effects of COVID-19 on population health [3,16,17,19]. These are powerful sources of real-time data that allow for estimation of the direct impact of COVID-19 on health service access and demand. However, they provide poor quality and limited data for accurately assessing the population-level effects of the pandemic and associated inequalities. Yet there is increasing evidence that COVID-19 is intensifying inequalities in maternal and child health, especially in communities that are already disadvantaged [11,20]. Routine health information systems data reflect services offered from the supply side and must be supplemented with a deeper understanding of the impact among those who are unable to access services, behaviour changes, and constraints. Furthermore, measuring the indirect effects of COVID-19 using routine health information systems data must correct for the possible under-reporting and inaccuracies caused by COVID-19 and pre-existing inconsistencies in these data systems. Meanwhile, prior modelling studies have been based on assumptions and scenarios which imply measurement uncertainties and the challenges of considering country-specific contexts, internal inequalities, and underlying factors.

Therefore, we aimed to assess the ‘true’ indirect effects of the COVID-19 pandemic on maternal, newborn, and child health at the population level in Burkina Faso, based on survey delivered prior to and during the COVID-19 pandemic. We aim to overcome the stated limitations of other methods and produce empirical evidence that may provide strategic orientation on appropriate mitigation and resiliency measures to implement in a pandemic context, accounting for population needs and existing socio-economic inequalities.

## METHODS

### Study setting

The first cases of COVID-19 infections were officially registered in Burkina Faso on 9 March 2020. There were 22 056 cumulative cases and 396 cumulative deaths as of 26 April 2023 [21]. These numbers, which are based primarily on the diagnosis of suspected cases and surveillance of infection in travellers rather than mass screening, were probably underestimated. A seroprevalence study of COVID-19 indicated that about 90% of the national population had at least one COVID-19 antibody acquired either through natural infection or vaccination. The national coverage of COVID-19 vaccination was estimated at 12% [22–24]. The country established nationwide sanitary and containment measures and policies early in the pandemic outbreak in March 2020. These included temporary shutdown of cities, curfew, closing borders, closing schools, and the shutdown of public places such as markets and places of worship. Additional measures involved banning social gatherings, enforcing physical distancing, and mandating the wearing of facemasks.

We conducted this study in Kadiogo and Boulkiemdé provinces. Several factors lay behind this choice. First, these provinces were priority areas for some United Nations organisations and non-government organisations. They met the criteria for an urban-rural comparison design and were not affected by the conflicts and security issues in Burkina Faso. The national capital, Ouagadougou, is located in the province of Kadiogo, which had a population of 3 030 384 inhabitants in 2019, representing 15% of the national population at the time [25]. This province, predominantly urban, is in the region of Centre, which experienced the highest COVID-19 burden out of the 13 regions, with 60% of cases and 59% of deaths of the country's total number over the 2020–21 period. The second most affected region, Haut-Bassins, accounted for 16% of cases and 21% of deaths. There were 416 health facilities including 10 hospitals in 2021 in the region of Centre [23,24]. The province of Boulkiemdé is in the Centre-Ouest region which was among the less affected regions, with about 1% of the total number of cases and deaths. The Centre-Ouest region had 281 health facilities, including five hospitals [23,24]. The 689 709 inhabitants in Boulkiemdé province were predominantly rural, less educated, and represented 3.4% of the total population [25].

### Study design and data

We used an observational design by collecting population-level data before and during the pandemic across urban and rural strata to assess the association between exposure to the pandemic and coverage of maternal and child health interventions. We carried out two cross-sectional household surveys in both provinces. Immediately before the pandemic outbreak (February to March 2020), we conducted a large household survey as part of the Real Accountability: Data and Analysis for Results (RADAR) project [26]. The survey included key maternal and child coverage indicators covering both urban and rural strata, and provided baseline data for the period before COVID-19. We carried out a second household survey (COVID survey) based on the same methodology two years into the pandemic (May to June 2022) for COVID-19-specific purposes.

The study design was similar for both surveys and data were collected in the same clusters before and during the pandemic. We implemented a multistage cluster sampling design stratified by residence area (50% rural and 50% urban). The target sample was 3375 households across 150 clusters per survey and equally distributed by stratum. The sample size was based on the assumptions of 50%-point estimates which is the most conservative estimate, 2% of non-response rate, 1.6 to 2.8 design effect for the main indicators. We inflated the sample size by 10% in the rural stratum and by 15% in the urban stratum to account for the proportion of vacant dwellings and non-residential structures identified as residential dwellings during the mapping. We used a geographic information system-based probability sampling method for residential dwellings identified as household units. This consisted of identifying and enumerating potential residential structures from freely available satellite images of the selected clusters (i.e. Google Maps, Open Street maps and Microsoft Bing aerial maps) [27–29]. We digitised the sampled clusters using sketched base maps from the national census and satellite images. After georeferencing all potential residential structures within each cluster, we created a sampling frame and sampled potential residential structures based on systematic random sampling. Interviewers used a navigation application [30] and satellite images to locate sampled structures and households in the field. The sample precision was 3–6 percentage points for key indicators and allowed to detect a difference of 4–12 percentage points with a power of 80% between the two data points.

We used three separate survey questionnaires (i.e. household questionnaire, woman’s questionnaire, and under-five children questionnaire) to collect socio-demographic information, COVID-19 knowledge and vaccination status, food security, and safety net. We also collected data on reproductive, maternal and child health services before and during the pandemic. This study focussed on maternal and child health interventions which include antenatal care (ANC), delivery care, postnatal care (PNC), and child routine vaccination and care seeking. Over 90% of households, eligible women, and children were successfully interviewed. The interview response rates and population samples were similar across the two surveys (Table S1 in the **Online Supplementary Document**).

### Analysis

**Figure 1:**
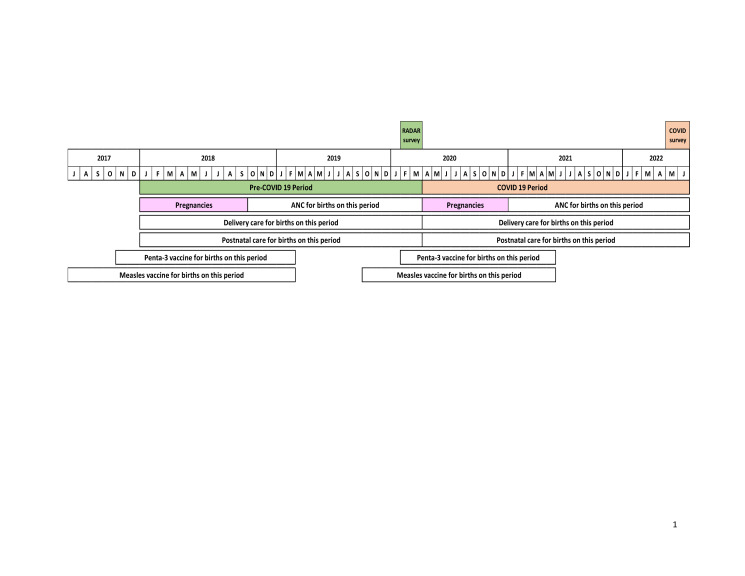
Data collection periods and reference periods for calculations of maternal, newborn, and child health coverage indicators.

We defined coverage as the proportion of individuals in need of a health service or intervention who received it. We assessed the impact of COVID-19 on coverage outcomes by comparing pre-pandemic period coverage estimates with estimates during the pandemic. Given that the pandemic was nationwide, we were unable to identify comparison areas for the analysis. We set up reference periods of maternal and child health indicators to examine the exposure time during the COVID-19 pandemic (**Figure 1**). In addition to the changes in service coverage over time (before vs. during COVID-19), we assessed the difference according to the place of residence (i.e. urban vs. rural). Furthermore, we examined the change in the coverage of the content of services provided to women and children. We identified service content interventions, which were used as an alternative to service quality, according to global guidelines and quality of care measurement [31,32]. Each health service comprises specific interventions for which we analysed the service coverage and content of care provided to women.

ANC coverage interventions included at least one ANC visit by a skilled provider, at least four visits by any provider during pregnancy, the source ANC (i.e. public vs. private facility), and whether the partner/spouse was present during the ANC visit with the health provider. The antenatal content items comprise blood pressure checks, blood and urine sample testing, counselling about pregnancy complications and nutrition, receipt of intermittent preventive treatment for malaria, iron/folic acid supplementation, and tetanus toxoid vaccine uptake. We estimated the coverage of institutional delivery, delivery by a skilled attendant, caesarean section rates, and the proportion of partners who accompanied the women to deliver and were present in the delivery room. We also calculated the proportion of newborns breastfed within one hour after delivery. We analysed any changes in women’s plans regarding the place of delivery. Additionally, we estimated the proportion of PNC for mothers and babies within two days after delivery and the source of PNC. The content of interventions for the mother PNC includes blood pressure and temperature checks, counselling about vaginal bleeding, and family planning. The practice of the skin-to-skin method, examination of the umbilical cord, non-application of harmful substances on the cord, counselling about newborn danger signs, counselling about breastfeeding, and observation of newborn’s first breastfeeding by a health provider were the intervention items for babies PNC.

We analysed the proportion of women who missed or delayed ANC and PNC along with the reasons and barriers to accessing care during the COVID-19 period. We examined the effects of COVID-19 on child routine vaccination including the coverage of diphtheria, pertussis, and tetanus (DPT)/pentavalent (1, 2, and 3 doses) and the first dose of measles vaccine, the percentage of vaccination dropout between DPT1 and DPT3 vaccination. We also assessed changes in timely vaccination of DPT1 (6–11 weeks of birth), DPT2 (10–19 weeks), DPT3 (14–24 weeks), and measles (6–12 months) defined according to the country vaccination schedule and the WHO recommendations for routine vaccination [33]. We estimated the proportion of children who experienced missed or delayed routine vaccinations and delays in seeking care for illnesses.

We carried out the analyses accounting for the survey's complex sampling design, clustering, and stratification. Additionally, we calculated standard errors and 95% confidence intervals (CIs) for all the indicators to assess whether the differences were statistically significant. The statistical analyses were done using Stata, version 16.1 (StataCorp, Texas, USA).

## RESULTS

### Effects of the COVID-19 pandemic on coverage and content of ANC service

**Figure 2:**
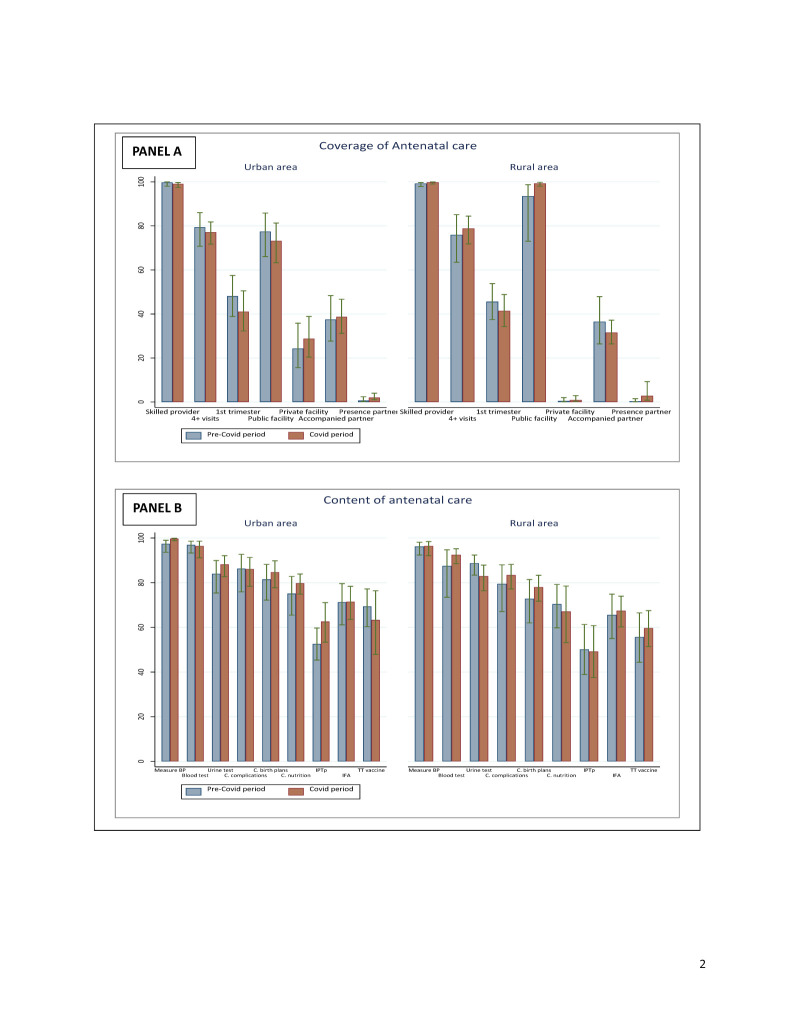
Coverage of ANC visit and content by place of residence before and during the COVID-19 pandemic. **Panel A. **Coverage of ANC. **Panel B.** Content of ANC. ANC – antenatal care

A high proportion of women sought at least one ANC from a skilled provider in both study provinces. There were no changes over time since the estimates prior to and during the were very high and similar (90%) across both areas. The proportion of women who attended at least four ANC visits during pregnancy dropped by 2 percentage points in the urban province. We did not find significant changes in the proportions of ANC initiation during the first trimester of pregnancy in both the urban and rural provinces. Similarly, the proportions of women who attended any ANC visit in a private or public health facility did not change over time in both provinces. There were no changes in the proportion of women accompanied by their partner to a facility for an ANC visit, and whose partner was present in the room or where the ANC clinical encounter took place (**Figure 2**, Panel A).

In the COVID-19 period survey, only 1.1% (95% CI = 0.6–1.8) of women in urban areas and 2.5% (95% CI = 1.0–6.0) of women in rural areas reported missing or delaying ANC due to COVID-19. Similar proportions of women experienced barriers in accessing ANC because of COVID-19; the main barriers and reasons were the congestion of health facilities (54.5%; 95% CI = 30.3–76.8) in urban areas and the fear of contracting COVID-19 (50.8%; 95% CI = 14.4–86.4)in rural areas (Table S2 in the **Online Supplementary Document**).

Regarding the content of ANC, there was no significant change over time in the coverage of interventions such as blood pressure measurement, blood and urine sample testing, counselling of women on pregnancy complications and nutrition, receipt of intermittent preventive treatment for malaria during pregnancy, iron/folic acid supplementation, and tetanus toxoid vaccination (**Figure 2**, Panel B).

### Effects of the COVID-19 pandemic on coverage of delivery care service

**Figure 3:**
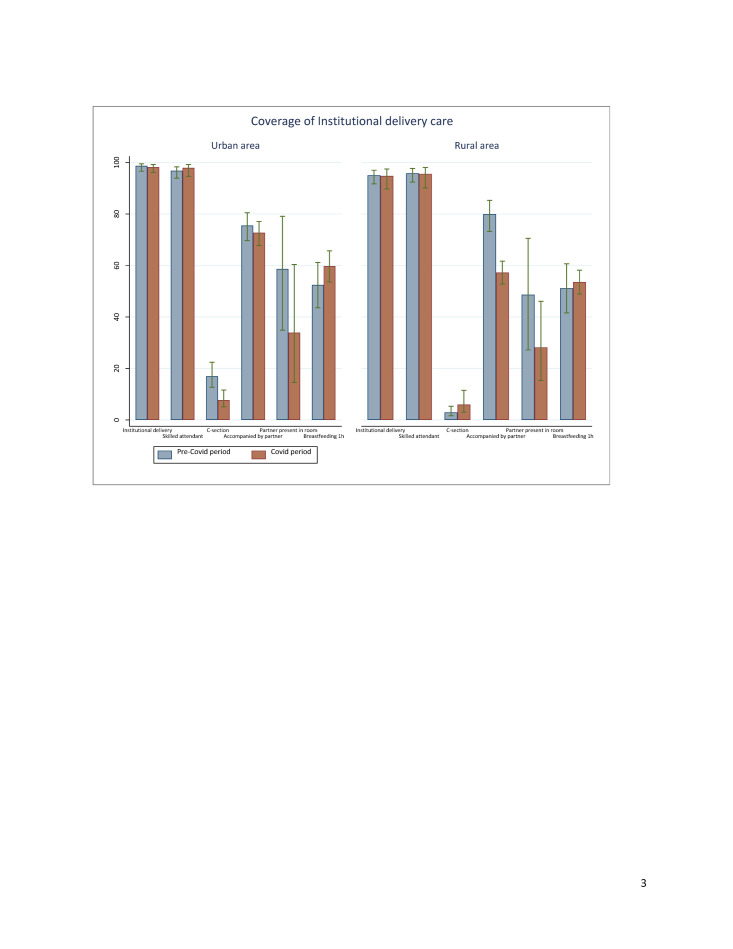
Coverage of delivery care by place of residence before and during the COVID-19 pandemic.

The coverage estimates of institutional delivery before COVID-19 (rural area: 95.0%; 95% CI = 91.7–97.0; urban area: 98.7%; 95% CI = 96.6–99.5) and skilled attendant at birth (rural area: 95.8%; 95% CI = 92.4–97.7; urban area: 96.8%; 95% CI = 93.9–98.3) were very high in both areas. Those proportions remain practically unchanged during the COVID-19 period. There was a significant drop in the percentage of women (22 percentage points) accompanied by partners to deliver in a facility in rural areas, but the change was not statistically significant in urban areas. Regarding the presence of partners in the delivery room, there were no differences between the proportions before the pandemic and the proportions during the pandemic. No significant change was observed in terms of coverage of early initiation of breastfeeding within one hour of birth. No women have changed their original plan regarding the place of delivery in rural areas, while only 2.9% (95% CI = 0.5–15) of urban women reported changing their place of birth because of the pandemic (Table S3 in the **Online Supplementary Document**). There was a significant decline in the proportion of women who gave birth by caesarean section in urban areas. The proportion of caesarean section deliveries dropped from 17% (95% CI = 12.7–22.4) to 7.7% (95% CI = 5.1–11.6) during the pandemic (**Figure 3**).

### Effects of the COVID-19 pandemic on coverage and content of PNC service for mothers and babies

**Figure 4:**
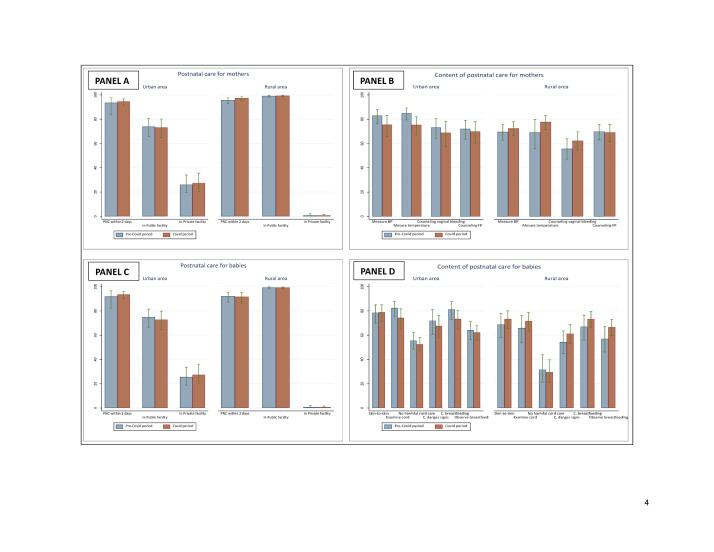
Coverage of PNC visits and content for mothers and babies by place of residence before and during COVID-19 pandemic. **Panel A.** Coverage of PNC for mothers. **Panel B.** Content of PNC for mothers. **Panel C.** Coverage of PNC for babies. **Panel D.** Content of PNC for babies. PNC – postnatal care.

The coverage of PNC interventions for mothers and babies was very high in the two areas (**Figure 4**, Panels A and C). Over 94% of women and 92% of babies attended a postnatal visit within the two days after birth primarily in public health facilities. The comparison of pre-pandemic and pandemic estimates showed no significant changes. We noted marginal declines in the content of PNC for both mothers and babies in urban areas, but the changes were not statistically significant (**Figure 4**, Panels B and D). Less than 1% of the women reported having missed or delayed PNC or experienced barriers in accessing PNC because of COVID-19 (Table S4 in the **Online Supplementary Document**).

### Effects of the COVID-19 pandemic on child routine vaccination and care seeking

The COVID-19 pandemic did not adversely affect vaccine coverage among children. There was no difference between the pre-COVID-19 and COVID-19 period for all vaccine coverages, except for DPT3 coverage, which increased significantly from 73.3% (95% CI = 66.0–79.6) to 88.3% (95% CI = 83.1–92.1) in urban areas (**Table 1**). Consequently, the vaccination dropout rate (i.e. the proportion of children who received DPT1 but not DPT3), significantly dropped from 16% (95% CI = 11.3–22.0) to 3.6% (95% CI = 1.7–7.6). There were no adverse effects on the timeliness of routine vaccines for children.

**Table 1:** Coverage of child routine vaccination

	Urban area		Rural area	
	**Pre-COVID-19**	**COVID-19 period**	**Pre-COVID-19**	**COVID-19 period**
**Type of vaccine**	**% (95% CI)**	**% (95% CI)**	**% (95% CI)**	**% (95% CI)**
DPT1/penta1 vaccine	94.5 (88.7–97.4)	91.2 (85.6–94.8)	94.9 (90.6–97.3)	88.3 (80.2–93.3)
DPT2/penta2 vaccine	86.5 (80.6–90.8)	90.5 (85.2–94.0)	90.6 (84.1–94.7)	88.6 (81.9–93.0)
DTP3/penta3 vaccine	73.3 (66.0–79.6)	88.3 (83.1–92.1)	84.9 (76.8–90.6)	87.1 (80.3–91.8)
Measles vaccine	83.5 (73.9–90.0)	83.4 (80.0–86.3)	81.3 (71.0–88.5)	83.6 (77.1–88.5)
Dropout between DPT1 and DPT3	16.0 (11.3–22.0)	3.6 (1.7–7.6)	6.3 (3.1–12.5)	3.5 (1.9–6.4)
DPT1 vaccine taken on time (6–11 weeks)	55.0 (45.2–64.4)	58.7 (50.6–66.3)	67.4 (58.0–75.6)	52.5 (41.3–63.4)
DPT2 vaccine taken on time (10–19 weeks)	63.8 (55.1–71.6)	68.6 (62.1–74.5)	67.4 (59.2–74.7)	63.8 (55.3–71.5)
DPT3 vaccine taken on time (14–24 weeks)	49.1 (41.6–56.8)	65.2 (58.6–71.2)	65.5 (55.2–74.5)	58.9 (50.4–67.0)
Measles vaccine taken on time (6–12 months)	44.9 (36.8–53.3)	56.4 (48.1–64.4)	49.2 (38.1–60.5)	51.4 (42.1–60.6)

DPT – diphtheria, pertussis, and tetanus

Mothers and caretakers reported that 6.2% (95% CI = 3.2–11.7) of urban and 2.8% (95% CI = 1.6–4.8) of rural children had missed a vaccine or that the vaccine uptake was delayed for COVID-19-related reasons (Table S5 in the **Online Supplementary Document**). The main barriers and reasons were the disruption of health services, fear of getting COVID-19, and mobility restrictions in urban areas. The disruption of health services, fear of getting COVID-19, and lack of transport were the main reasons in rural areas. Concerning sick children, 2.3% (95% CI = 1.4–3.8) in urban and 3.1% (95% CI = 1.9–5.1) in rural areas did not seek care or care-seeking were delayed due to COVID-19 (Table S6 in the **Online Supplementary Document**).

## DISCUSSION

The COVID-19 outbreak raised major concerns about the stronger reversals in hard-earned gains in reproductive, maternal, newborn, and child health and nutrition services coverage in LMICs, specifically in sub-Saharan Africa [1–6]. That was based on the fragile political and socioeconomic contexts, experiences from prior pandemics such as Ebola and HIV/AIDS, and poor health conditions and preparedness of health systems to respond to COVID-19. Our study, based on empirical data from surveys conducted immediately before the pandemic and two years during the pandemic in two urban and rural provinces in Burkina Faso, demonstrates that these concerns did not materialise, at least not at the scale projected.

The coverage of ANC, institutional delivery, skilled attendant at birth, PNC for mothers and babies within two days of birth, and DPT1 vaccination was very high before and during the COVID-19 pandemic. Overall, there were no dramatic changes over time in terms of maternal and child health intervention coverage and quality of care in both urban and rural provinces studied. Very few women have missed or delayed ANC and PNC visits because of difficulties and barriers related to the COVID-19 pandemic. The few who reported difficulties stated the congestion of health facilities and the fear of getting COVID-19 as the main reasons. These reasons have also been mentioned as barriers to care-seeking in other settings [11,34,35]. The findings are consistent with evidence from other studies in sub-Saharan African countries. An assessment of the effects of the pandemic from routine health data showed non-significant changes in ANC coverage at the national level in Kenya [36]. In Ethiopia, there were no disruptions in ANC coverage intervention, and marginal effects were observed on skilled birth attendant coverage [36]. Several individual countries and multi-country studies showed similar conclusions, mixed findings, or small adverse effects of the pandemic on maternal and newborn health [4,16,17].

There were no major disruptions in birth preparedness, since most women declared that they delivered in the facility identified in their original plan. Conversely, there was a large drop in the proportion of women accompanied by their partners to the health facility to deliver, particularly in rural areas. In the context of our study, it is not unexpected that very few partners accompany their pregnant wife for delivery. However, the sharp drop in men’s presence at childbirth during the COVID-19 period may be interpreted as a potential consequence of the pandemic. The fear of COVID-19 and the enforced social distancing may prevent partners from accompanying their spouses to the facility. Political and containment measures were indeed major barriers to care-seeking and health service use during the pandemic [11].

The large drop of over 50% in the caesarean section rate during the pandemic in urban areas specifically was a remarkable finding that may be explained by the reduction of medically unnecessary caesarean section delivery and health workforce task shifting during the pandemic. Before the pandemic, caesarean section delivery rates had rapidly and continuously increased following the implementation of a free maternal health care policy in 2016 in Burkina Faso [37,38]. However, a study based on the WHO-recommended Robson's classification for audit and classification of caesarean section in Ouagadougou, the capital city, suggested that a substantial proportion of caesarean section deliveries were unnecessary and found that about one-third of overall caesarean section deliveries were performed in low-risk women [37]. Furthermore, the findings from a cluster-randomised controlled trial to reduce unnecessary caesarean section deliveries in Burkina Faso showed a large drop from 18.9% to 6.5% of caesarean section rate in the intervention arm, flagging the issue of unnecessary and elective caesarean section being performed in low-risk women [39]. Indeed, there is evidence that one-quarter of caesarean section deliveries were without emergency needs or medical indication [40,41].

Following the pandemic, we assume that health service efforts were first and foremost directed towards emergency caesarean section deliveries vs. elective cases given health facilities congestion and the shortage of health staff dealing with the pandemic as a priority. There were no relationships between the decline in caesarean section deliveries and the proportions of institutional deliveries as the coverage of the latter did not decline during the pandemic. There was likely less demand for unnecessary caesarean deliveries given the need for hospitalisation and a possible longer period of exposure to COVID-19 during the hospital stay. In many countries, the health workforce was reallocated for patient testing, triage, treatment, vaccination, counseling, and other medical responses to COVID-19. [42,43]. The fear of getting COVID-19 after caesarean delivery may have resulted in reduced elective caesarean sections and subsequently the overall caesarean section rate. Therefore, caesarean section deliveries during the pandemic may be those for emergency needs or medical indications as required by the WHO. Indeed, the WHO pointed out the rise of medically unnecessary and unjustified caesarean section rates as a public health concern and the association with increased maternal and perinatal morbidity [44].

Our findings did not show major disruptions to child vaccination and general health care seeking from a health facility. However, there were drops regarding the timing of DPT1, DPT2, and DPT3 vaccine coverage during the COVID-19 period in rural areas. The large confidence intervals around the estimates made it difficult to conclude about the statistical significance. Furthermore, the increased coverage of DPT1 and DPT3 vaccines in urban areas may be seen as an indication of the health system's resilience and the population’s awareness about child routine vaccine uptake and beneficial effects.

There were a few limitations to this study. First, it was challenging to restrict the analysis to the periods of higher stringency of containment measures (March to August 2020) for some indicators due to sample size issues. Another limitation was the retrospective nature of the questions which may be affected by memory or recall bias. A third limitation was the representativeness of the study which relies on two regions out of the thirteen regions in Burkina Faso, making it difficult to generalise our findings nationally. However, these two provinces were interesting case studies for several reasons. First, the Kadiogo province is the capital city which had recorded the highest COVID-19 morbidity and mortality burden. This is a predominantly urban area with the highest stringency in containment measures. Therefore, stronger disruptions of health services and population health outcomes were expected. In contrast, the province of Boulkiemdé is predominantly rural and was among the provinces with the lower number of cases and deaths of COVID-19. These two provinces were also selected because of the availability of baseline data sets from a household survey conducted immediately before the pandemic.

Concerning the strengths of the study, we should first note the nature of the data we used. There were very few community-based sources of data available to assess the effects of COVID-19 on the coverage of maternal, newborn, and child health according to pre-pandemic and pandemic period design. Both baseline and end-line surveys were conducted during the same season to avoid any bias due to the timing of the surveys. Furthermore, the timing of the surveys had no or little influence on the health intervention indicators included in our study. Most studies relied on routine health facility data and modelling methods because of the challenge of empirical baseline data and the difficulty of conducting direct in-person interviews during the pandemic [1–4,16,17,19,36,45]. Modelling methods do not provide an accurate representation of the effects of COVID-19. They are based on assumptions and scenarios with large measurement uncertainties and generally do not assess subnational inequalities. On the other hand, routine health facility data suffer from data quality and denominator issues and are limited in measuring health intervention coverage [46,47]. Indeed, there are completeness of reporting issues and denominator issues as data don’t include all facilities data and all individuals in need of health intervention, only individuals visiting health facilities are considered. It is also challenging to assess socioeconomic inequalities and underlying factors using routine health facility data and modelling estimates, though the pandemic is intensifying maternal and child health inequalities in disadvantaged communities [11,20]. In contrast, household survey data, used in our study, are ideal sources to measure the coverage of health interventions. They provide a complete and accurate understanding of the population-level impacts of the pandemic and related inequalities. They are also based on a rigorous sampling method, unlike phone-based surveys which were another source of data to assess the effects of the COVID-19 pandemic [48–51]. Another strength of the study was the comparative perspective of the effects of the pandemic between the urban and rural areas. These are different geographical and socioeconomic contexts which have experienced variable exposure to COVID-19 and associated political and emergency measures.

## CONCLUSIONS

Contrary to the early warnings, Burkina Faso experienced resilience to mitigate the negative impacts of COVID-19 on the coverage of maternal and newborn interventions. Although the coverage and content of maternal and newborn services remained stable, there was no significant improvement observed in most indicators. It is also unlikely that the pandemic has slowed down the increasing trends in maternal, newborn, and child health intervention coverage tendency and progress observed before the pandemic, given the small proportion of women who reported changes in their care-seeking and childbirth plans during the pandemic. This country-specific context study offers a nuanced perspective on the effects of COVID-19, challenging the alarmist forecasts. Additionally, the findings may be relevant to other LMICs as they point out the role of health systems resilience and the importance of equity in health service access to mitigate the effects of pandemics in resource-limited settings.

## Additional material


Online Supplementary Document
