# Supplementary material for: Effects of the COVID-19 pandemic on maternal, newborn, and child health service coverage in Burkina Faso
Source: J Glob Health. 2024 Dec 20;14:05037. doi: 10.7189/jogh.14.05037 (PMC11658717; doi:10.7189/jogh.14.05037)
Supplement: Online Supplementary Document [file jogh-14-05037-s001.pdf]

Supplementary Tables

Supplementary Table S1. Study sample and interview completeness

	Pre-Covid Survey		Covid Period Survey	
**Results**	Urban	Rural	Urban	Rural
**Household interviews**				
Households selected	1449	1466	1561	1471
Households surveyed	1316	1421	1496	1419
Response rate (%)	90.8	96.9	95.8	96.5
**Interviews of women aged 15-49**				
Eligible women	2132	2719	2571	2322
Women surveyed	1960	2540	2449	2076
Response rate (%)	91.9	93.4	95.3	89.4
**Interviews of children 0-59 months**				
Children eligible	955	1914	1226	1582
Children surveyed	908	1826	1201	1477
Response rate (%)	95.1	95.4	98.0	93.4

Supplementary Table S2. Distribution of women who experienced any barriers in accessing antenatal care during the Covid-19 period

**Indicators**	**Urban**		**Rural**	
	**Percentage**	**95% CI**	**Percentage**	**95% CI**
Proportion of women who missed or delayed antenatal care because of Covid-19	1.1	[0.6-1.8]	2.5	[1.0-6.0]
Proportion of women who experienced barriers to accessing antenatal care because of Covid-19	1.0	[0.6-1.7]	1.8	[0.7-4.3]
**Among women who experienced barriers to accessing antenatal care, proportion of those who cited the following barriers:**				
Lack of money for services	11.1	[2.8-35.3]	16.7	[8.0-31.7]
No medical staff	0.0		11.2	[2.7-35.9]
Medical establishment was saturated	54.5	[30.3-76.8]	14.3	[4.0-40.0]
Medical establishment was closed	5.3	[1.0-23.3]	13.2	[3.6-38.3]
Medical facility didn't have enough supplies or tests	18.5	[4.9-49.9]	0.0	
Fear / worry of catching Covid-19	8.4	[2.3-26.6]	50.8	[14.4-86.4]
Restrictions (stay-at-home orders) / curfew	4.1	[0.5-25.2]	0.0	
Lack of transport	0.0		4.4	[1.1-16.0]
Responsibilities of the service provider	3.7	[0.8-16.1]	2.9	[0.5-14.7]
Service provider delayed/cancelled visit	6.3	[1.4-24.2]	7.1	[1.5-28.0]
Other	2.2	[0.3-16.1]	7.2	[1.3-31.5]
**N**	**23**		**33**	

Supplementary Table S3. Distribution of women who have changed their place of birth due to Covid-19

Change of place of birth	Urban			Rural		
	Percentage	95% CI	N	Percentage	95% CI	N
No	97.1	[85.0-99.5]	77	100.0	-	112
Yes	2.9	[0.5-15.0]	2	0.0	-	0
**Total**	**100**		**79**	**100**		**112**

Supplementary Table S4. Distribution of women who experienced any barriers in accessing postnatal care during the Covid-19 period

Indicators	Urban		Rural	
	Percentage	95% CI	Percentage	95% CI
Proportion of women who missed or delayed postnatal care because of Covid-19	0.6	[0.3-1.3]	0.9	[0.4-1.9]
Proportion of women who experienced barriers in accessing postnatal care because of Covid-19	0.1	[0.0-0.4]	0.4	[0.2-0.9]
**Among women who experienced barriers to accessing postnatal care. proportion of those who cited the following barriers**				
No medical staff	0.0		39.0	[7.7-83.1]
Medical establishment was saturated	32.7	[3.5-86.6]	0.0	
Medical establishment was closed	0.0		20.7	[4.4-60.1]
Medical facility didn't have enough supplies or tests	13.2	[2.5-47.7]	3.2	[0.3-26.1]
Fear / worry of catching Covid-19	0.0		29.4	[7.3-69.0]
Restrictions (stay-at-home orders) / curfew	14.8	[0.9-76.9]	11.3	[1.8-47.6]
Lack of transport	0.0		15.4	[2.4-57.5]
Service provider delayed/cancelled visit	20.9	[1.3-84.0]	0.0	
Other	33.1	[4.7-83.4]	5.5	[0.5-40.3]
**N**	**5**		**12**	

Supplementary Table S5. Distribution of children whose routine vaccination was delayed/missed due to Covid-19

Indicators	Urban			Rural		
	Percentage	95% CI	N	Percentage	95% CI	N
Children whose vaccination was delayed or missed due to Covid-19	6.2	[3.2-11.7]	674	2.8	[1.6-4.8]	854
**Reasons for delayed or missed vaccination**						
Fear of catching Covid-19	25.7	[15.5-39.4]	34	20.7	[8.8-41.3]	27
Disruption of health services	41.0	[27.0-56.7]	34	25.8	[10.0-52.0]	27
Mobility restrictions	19.9	[9.9-35.8]	34	6.4	[0.8-37.0]	27
Lack of transport	0.0		34	20.5	[3.4-65.5]	27
Cost/affordability	3.9	[0.4-26.7]	34	1.4	[0.2-10.5]	27
No reason	20.1	[6.9-45.9]	34	28.6	[11.6-54.9]	27
Other	0.8	[0.1-6.4]	34	0.0		27

Supplementary Table S6. Distribution of whose care seeking was delayed/missed due to Covid-19

Indicators	Urban			Rural		
	Percentage	95% CI	N	Percentage	95% CI	N
Children whose health care has been delayed or missed due to Covid-19 (March/September 2020)	2.3	[1.4-3.8]	785	3.1	[1.9-5.1]	1020
Children whose health care was delayed or missed because of Covid-19 (last 2 years)	1.5	[0.8-2.9]	1192	2.2	[1.1-4.2]	1467
**Reasons for delays or shortcomings in the child's health care**						
Fear of catching Covid-19	43.0	[19.7-69.8]	23	58.5	[31.9-80.9]	44
Disruption of health services	23.4	[8.6-49.6]	23	25.2	[10.4-49.5]	44
Mobility restrictions	15.4	[3.5-48.0]	23	11.1	[4.2-26.3]	44
Lack of transport	5.3	[1.2-21.3]	23	7.6	[2.0-24.8]	44
Self-isolation due to suspicion of Covid-19	3.2	[0.4-22.1]	23	0.8	[0.1-6.4]	44
Cost/affordability	19.4	[5.8-48.9]	23	5.9	[1.2-23.6]	44
Other	0.0		23	5.9	[1.7-18.5]	44
